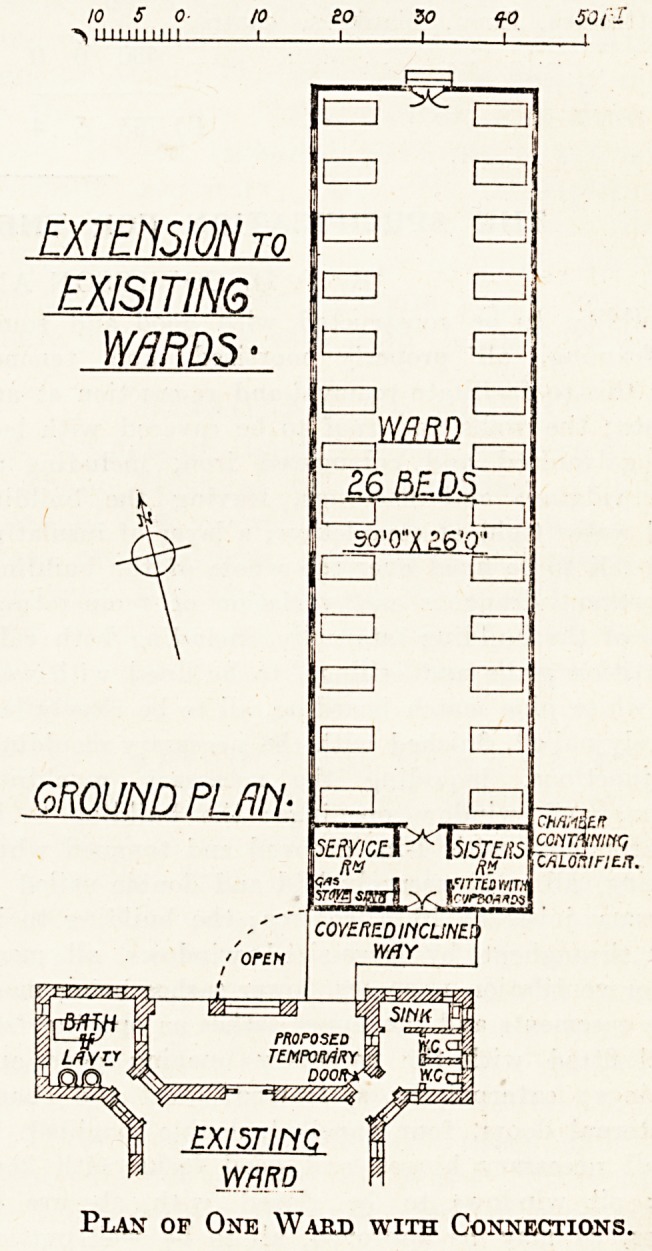# The Special Wards for Wounded Soldiers

**Published:** 1914-10-31

**Authors:** 


### THE ROYAL INFIRMARY, NEWCASTLE-ON-TYNE.

### The Special Wards for Wounded Soldiers.

In The Hospital of September 12 last, page 655, we published an advance Note on the temporary wards for the wounded at this infirmary. To-day we have the pleasure to publish a description of these wards, with a plan, for which we are indebted to Mr. Roden Orde, the house governor and secretary of the Newcastle Royal Victoria Infirmary. The plan adopted is a novelty; it brings into notice a Scotch firm, Messrs. F. D. Cowieson and Co., who have made temporary buildings, and those for hospital use a speciality, and we commend with pleasure the high testimony to their efficiency spontaneously given by Mr. Roden Orde. There is undoubtedly a great need in this country for thoroughly reliable and up-to-date facilities for this kind of work, in conjunction with a firm which can be trusted and absolutely relied upon to stick to its estimates and produce none but the best work. Mr. Orde has sent us the original specification for the new buildings at Newcastle, which we hope will prove of interest and value to those of our readers who have to face problems which have arisen or arise out of the war.

Directly the authorities found they required these extra wards the house governor got into communication with Messrs. Cowieson and Co., of Glasgow, a firm that has done work for the infirmary before. They put up a temporary building for the committee at Newcastle five years ago; at the end of four years they moved it from one position to another, where it still stands, and it is as good as new. The house governor explained to Cowiesons what was wanted, and they promptly let him have drawings. Luckily, Newcastle's wants were very simple, as the infirmary authorities wished to save every penny they could. At first they thought of putting temporary blocks between the Royal Infirmary pavilions in order to get access from the main corridor. The house governor proceeds: We felt, however, that in spite of difficulty of access anything was better than blocking up the spaces between the present wards. Finally, we came to the conclusion that it would be better to put up with inconvenience of access so long as we could secure plenty of air for the new buildings, without interfering with the supply of air to the old; so we determined to put our new wards up, practically as elongations of the old ones. As at the end of each of our wards there is a large glass door which acts as a ventilator and allows a view of the garden to the patients, we decided to stagger the new wards to one side. The new wards, therefore, interfere with the old ones very little indeed. Luckily, too, there is not much difference in the level, and the open gangway leading from the old to the new wards slopes only slightly upwards. We could have saved a good deal of this slope had we not been determined to have a good air space between the ground and the floor of the new building. This air-space is well ventilated by openings all round.

The new wards stand on creosoted timber foundations, laid on the earth. We had the ground thoroughly covered with lime and levelled. The sides of the building from ground level to eaves are of weather boarding; the roof is of corrugated iron. There is a layer of felt on the inside of the walls and roof and an air-space before you come to the match-boarding which lines the interior. At first we intended to have a flat ceiling, starting about 2 feet above where the roof begins to slope inwards, but as this would have reduced the air space, and as in these times every available inch of floor space will probably be made use of, we decided to have a sloped instead of a flat roof. Of course, this exposes the roof beams, but I think the balance is in favour of plenty of air. There are big doors at

Plan of One Ward with Connections.

either end of the wards in case the wards have to be hurriedly cleared. The floor is a plank floor; it is very well laid, and is to be covered with linoleum. There are three large ventilators in the roof, and perforations along the whole line of the ridge. The windows are French windows with hoppers above. The heating is by a double row of cast-iron pipes with three large radiators. These pipes are heated from two calorifiers, in chambers built at the side of the ward, supplied by steam from our boilers.

At the entrance to the ward there are two rooms, one of which will be used as a kitchen, where we are placing a sink and a gas-stove. The other must be used by the sister in charge, and we shall have to put up in it any cupboards required for ward use, linen cupboards, &c. In order to save cost we have determined to make the new wards use the bathrooms, bed-pan sinks, and lavatories of the old wards.

### Fifty-two Beds in Two Wards for £1,753.

The following is our bill of costs for two wards containing fifty-two beds:—

| | £ | s. | d. |
|---|---|---|---|
| Building ... ... ... ... ... ... | 935 | 0 | 0 |
| Foundations ... ... .. ... | 30 | 0 | 0 |
| Lighting and telephone ... ... | 72 | 6 | 4 |
| Heating ... ... ... ... ... | 198 | 0 | 0 |
| Drainage ... ... ... ... ... | 38 | 0 | 0 |
| Furnishing with bedsteads, lockers, mattresses, linen, blankets, chairs, etc. ... ... ... ... ... | 480 | 0 | 0 |
| | £1,753 | 6 | 4 |

## Figures and Tables

**Figure f1:**